# Numerically optimized radiofrequency pulses for robust and low-power cardiovascular T_2 _preparation at 3T

**DOI:** 10.1186/1532-429X-16-S1-P41

**Published:** 2014-01-16

**Authors:** Ruud B van Heeswijk, Kieran R O'Brien, Jean Delacoste, Matthias Stuber

**Affiliations:** 1 Radiology, University Hospital (CHUV) and University of Lausanne (UNIL), Lausanne, Switzerland; 2 Center for Biomedical Imaging (CIBM), Lausanne, Switzerland; 3 Radiology, University of Geneva, Geneva, Switzerland

## Background

Cardiac magnetic resonance imaging (CMR) has been shown to benefit from the higher signal-to-noise ratio (SNR) and contrast-to-noise ratio (CNR) available at higher magnetic field strengths; however, in practice, CMR remains limited by the need for higher radiofrequency (RF) pulse power, which is in turn limited by the maximum specific absorption rate (SAR). For example at 3T, an adiabatic (robust to RF inhomogeneity ΔB1) T_2 _preparation (T_2_Prep, Nezafat et al., MagnResonMed2006) can usually only be combined with balanced steady-state free precession (bSSFP) acquisitions with low nutation angles, or is played out only once every several heartbeats. Thus the design of T_2_Prep adiabatic inversion pulses requires a compromise between pulse performance and the energy deposition. To overcome this SAR limitation on T_2_Prep, we therefore numerically optimized two hyperbolic secant (HSn; Silver et al. JMagnReson1984) RF pulses and tested their performance for T_2_Prep refocusing in CMR at 3T.

## Methods

A genetic algorithm based on Bloch equation simulations (Hurley et al., MagnResonMed2010) was used to numerically optimize standard adiabatic HS1 (higher power requirement and ΔB1 robustness) and HS8 pulses (lower power requirement and ΔB1 robustness) to generate Time-Resampled Frequency-Offset-Corrected Inversion (TR-FOCI) pulses with a duration of 12 ms and an inversion band of 300 mm, which should easily cover the cardiac anatomy. The minimum energy requirements for satisfactory T_2_Prep performance were assessed in agar-NiCl2 phantoms and 3 healthy volunteers with a 2D radial bSSFP imaging sequence (nutation angle 70°, matrix 256^2^, slice thickness 8 mm, lines per heartbeat 35) on a 3T clinical MR scanner (Skyra, Siemens) while monitoring SAR levels. The myocardium-to-blood CNR was calculated in both phantoms and volunteers and the minimum required pulse energy for constant CNR and absence of artifacts was compared.

## Results

**Figure 1:**
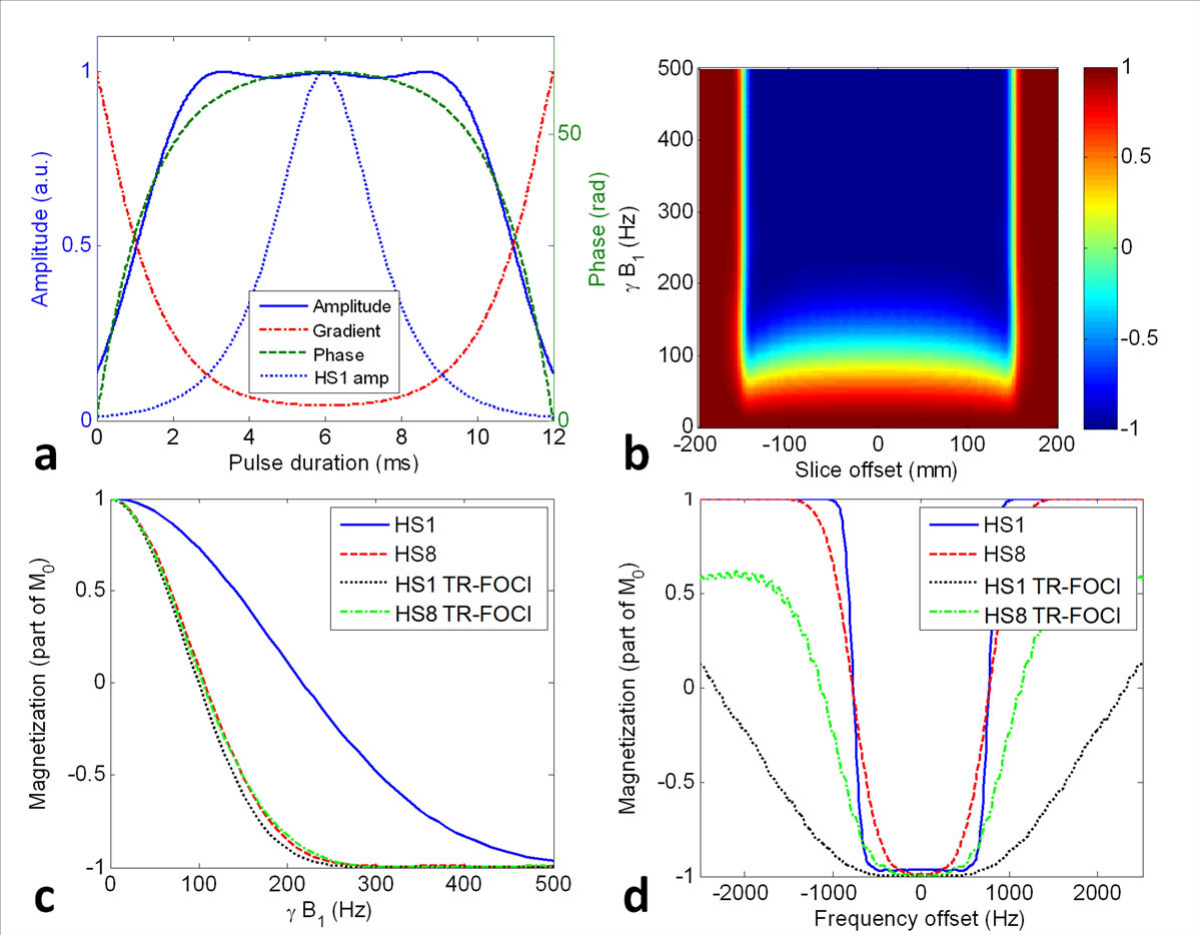
**Performance of the tailored pulses**. a) Example characteristics of the HS1-derived TR-FOCI pulse. Note that the optimized pulse is broad compared to the standard HS1 (dotted line). b) Slice-excitation profile of the same HS1-derived TR-FOCI pulse. Above a low pulse power of γB1≈200 Hz, the profile has a very sharp transition. c) On-resonance inversion power requirements of the standard and TR-FOCI pulses. Once the HS1 pulse is optimized as a TR-FOCI pulse, it requires power similar to the standard HS8 pulse. d) Robustness of the same pulses to B0 variability at pulse power γB1 = 500 Hz. While the poor original profile of the HS8 pulse is improved after optimization, it is especially the optimized HS1 that has improved robustness.

**Figure 2:**
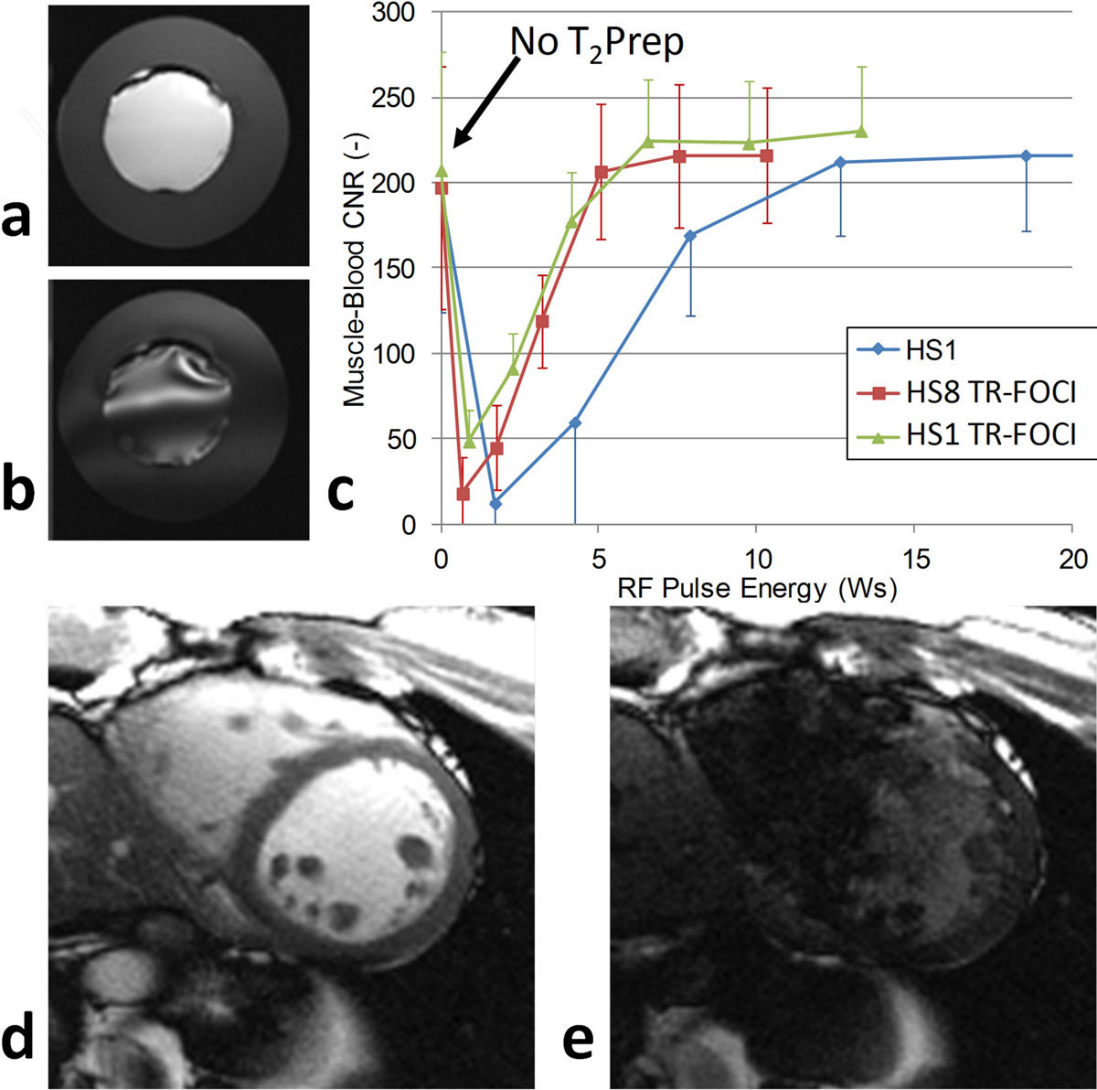
**Application of the TR-FOCI adiabatic pulses in the phantoms and volunteers**. a) Radial bSSFP image of the phantom with compartment T1 and T_2 _values that approach blood (bright center) and myocardium (darker outer layer). A HS8-derived TR-FOCI pulse with pulse energy 4Ws was used in the T_2_Prep. b) The same image, but with a 4Ws standard HS1 pulse. Distortions due to insufficient pulse energy can be clearly observed c) Contrast-to-noise ratio plots in the volunteers show that significantly less energy is required by the optimized pulses to obtain the same CNR. d) and e) In vivo human HS1-derived TR-FOCI image with T_2_Prep pulse energy ~4Ws: the superior performance of the optimized pulse compared to the standard HS1 pulse T_2_Prep can be clearly observed.

The optimized pulses demonstrated superior performance in the simulations compared to standard HSn pulses (Figure 1). The TR-FOCI pulses required 54% less power than the HS1 pulse to achieve artifact-free images and stable CNR (Figure 2), while images obtained with an HS8 pulse were never artifact-free. The optimized pulses needed roughly half the energy of the standard pulses, and the entire pulse sequence resulted in 20% less overall SAR deposition in the volunteers for artifact-free images with similar CNR as the original images.

## Conclusions

We successfully implemented numerically optimized adiabatic pulses and demonstrated that they required less power for similar performance to HSn pulses in a T_2_Prep, which critically enables the use of CMR with bSSFP and T_2_Prep at 3T.

